# Osmotic Demyelination Syndrome in Hyperosmolar Hyperglycemic State With Contrasting Initial Sodium Levels: A Report of Two Cases

**DOI:** 10.7759/cureus.88700

**Published:** 2025-07-24

**Authors:** Daigo Kasamatsu, Takefumi Tsunemitsu, Masaru Matsumoto, Takao Suzuki

**Affiliations:** 1 Department of Emergency and Critical Care Medicine, Hyogo Prefectural Amagasaki General Medical Center, Amagasaki, JPN; 2 Department of Preventive Services, School of Public Health, Kyoto University Graduate School of Medicine, Kyoto, JPN

**Keywords:** central pontine myelinolysis (cpm), diabetes mellitus, extrapontine myelinolysis (epm), hyperosmolar hyperglycemic state (hhs), osmotic demyelination syndrome (ods)

## Abstract

Osmotic demyelination syndrome (ODS) is typically associated with rapid correction of severe hyponatremia but is a rare complication of hyperosmolar hyperglycemic state (HHS). We describe two cases of ODS developing in HHS with contrasting initial sodium levels: one with profound hyponatremia (Na 112 mmol/L, corrected 167 mmol/L) and the other with hypernatremia (Na 149 mmol/L, corrected 177 mmol/L). Both patients exhibited severe hyperglycemia (2,398 and 1,277 mg/dL, respectively) and marked hyperosmolality (387 and 421 mOsm/kg) and were managed with cautious correction rates (1.2 and 1.0 mOsm/kg/hour). Despite this, each patient developed ODS diagnosed by MRI on days three and seven, respectively. Neurological function gradually improved in both, with final modified Rankin Scale scores of 2 and 1, respectively. To our knowledge, this is the first case report to directly compare hyponatremic and hypernatremic ODS in the context of HHS. Severe hyperosmolality can itself trigger ODS, regardless of measured and corrected sodium levels or the rate of correction. Clinicians should maintain a low threshold for timely MRI in HHS patients with persistent or unexplained neurological deficits.

## Introduction

Osmotic demyelination syndrome (ODS), a non-inflammatory disorder encompassing central pontine and extrapontine myelinolysis, is most commonly precipitated by the rapid correction of severe hyponatremia [1,2]. However, the occurrence of ODS in the setting of hyperosmolar hyperglycemic state (HHS) is rare. HHS is defined by severe hyperglycemia (plasma glucose > 600 mg/dL), effective serum osmolality > 320 mOsm/kg, and profound dehydration with little to no ketoacidosis [3]. A focused search on PubMed for English-language case reports and small series identified fewer than 30 unique patients worldwide who had ODS complicating HHS [4-11]. In these rare cases, several reports suggest that severe hyperosmolality itself, rather than its correction, is the primary trigger [12,13]. This process is thought to involve hypertonic stress-induced injury to the blood-brain barrier, ultimately leading to demyelination. Sodium abnormalities in HHS are complex: measured serum sodium may appear low (hyponatremia) on admission due to marked hyperglycemia, but after correction for hyperglycemia, a process known as "corrected sodium calculation," the true sodium level may fall into the hypernatremic range [14]. Both measured and corrected sodium values are clinically important and reflect different aspects of the osmotic burden on the brain. Here, we present, to the best of our knowledge, the first case report that directly compares two cases of ODS associated with HHS in the same hyperosmolar milieu: one with profound hyponatremia on admission (but hypernatremic after correction) and the other with hypernatremia. This direct comparison underscores the clinical diversity and diagnostic complexity of HHS-induced ODS and highlights the relevance of both measured and corrected sodium in risk assessment.

This article was previously presented as a meeting abstract at the 52nd Annual Meeting of the Japanese Society of Intensive Care Medicine on March 15, 2025.

## Case presentation

This is a retrospective case report of two patients with HHS-induced ODS who were treated at the Hyogo Prefectural Amagasaki General Medical Center between November 2021 and April 2022. Both cases were consecutive.

Case 1

A 58-year-old Japanese woman with untreated hyperglycemia and bipolar disorder presented with an altered mental status and a generalized seizure following a one-week period of poor appetite during which she had consumed 4 L of soft drinks daily. On arrival, the Glasgow Coma Scale (GCS) score was E1V1M1; vital signs showed tachycardia (119 beats/minute) and tachypnea (24 breaths/minute). Initial laboratory tests revealed severe hyperglycemia (2,398 mg/dL), profound hyponatremia (measured sodium 112 mmol/L), and hyperosmolality (387 mOsm/kg). After correction for hyperglycemia, her "corrected sodium" was calculated as 167 mmol/L, indicating hypernatremia. This discrepancy highlights the importance of considering both measured and corrected sodium in the evaluation of HHS. Venous blood gas analysis showed acidosis (pH 7.171), but the bicarbonate level was within the normal range (27.6 mmol/L), suggesting primarily respiratory rather than metabolic acidosis. Serum β-hydroxybutyrate was 2.8 mmol/L (2,781 μmol/L), which is below the commonly used threshold (3 mmol/L) for clinically significant ketoacidosis. Only mild urinary ketones were detected. These findings supported a diagnosis of HHS rather than diabetic ketoacidosis (DKA) or mixed HHS and DKA. All initial laboratory data for both cases are summarized in Table 1.

**Table 1:** Laboratory findings on admission for both cases Data for blood gas analysis in Case 1 are from venous blood; data for Case 2 are from arterial blood. Corrected sodium was calculated as: measured sodium + 2.4 × ((glucose – 100)/100), in accordance with published recommendations for severe hyperglycemia [15]. Normal ranges may vary between institutions. Abbreviations: HHS, hyperosmolar hyperglycemic state; DKA, diabetic ketoacidosis; WBC, white blood cell; PCO₂, partial pressure of carbon dioxide; HCO₃⁻, bicarbonate; CRP, C-reactive protein; BUN, blood urea nitrogen.

Laboratory Test (Units)	Case 1	Case 2	Normal Range
HHS/DKA-Related Tests			
Glucose (mg/dL)	2398	1277	73–109
Serum Osmolality (mOsm/L)	387	421	270–295
HbA1c (%)	13.8	11.8	4.9–6.0
β-Hydroxybutyrate (mmol/L)	2.8	11.8	< 0.5
Urine Ketones	1+	3+	Negative
Blood Gas and Acid-Base			
pH (Venous)	7.171	-	7.31–7.41
pH (Arterial)	-	7.033	7.35–7.45
PCO₂ (mmHg, Venous)	78.6	-	41–51
PCO₂ (mmHg, Arterial)	-	36.1	35–45
Bicarbonate (HCO₃⁻) (mmol/L)	27.6	9.2	22–26
Anion Gap (mmol/L)	22.4	30.8	10–14
Lactate (mmol/L)	6.6	2.4	0.5–1.6
Electrolytes and Minerals			
Sodium (mmol/L)	112	149	138–145
Corrected sodium (mmol/L)	167	177	－
Potassium (mmol/L)	5	4	3.6–4.8
Chloride (mmol/L)	62	109	101–108
Phosphorus (mg/dL)	12.1	9.8	2.7–4.8
Magnesium (mg/dL)	4	3.6	1.8–2.4
Other Laboratory Markers			
WBC (×10³/μL)	18.6	21.1	3.3–8.6
CRP (mg/dL)	8.88	3.02	0.00–0.14
BUN (mg/dL)	33.3	71	8–20
Creatinine (mg/dL)	3.25	3.41	0.46–0.79
Albumin (g/dL)	3.3	2.1	4.1–5.1
Creatine Kinase (U/L)	362	1853	41–153

**Figure 1:**
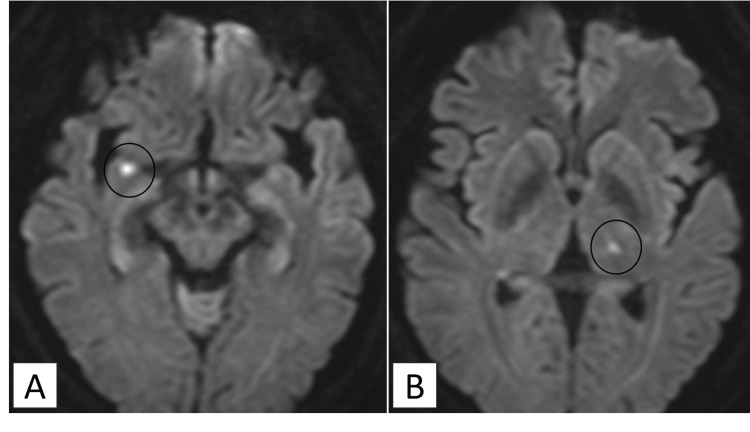
Case 1: Axial diffusion-weighted images (DWI) on day three Panel (A) shows a hyperintense lesion in the right lateral amygdala (circle). Panel (B) shows a hyperintense lesion in the left thalamus (circle).

The patient was intubated and admitted to the intensive care unit (ICU) for intravenous insulin and fluid resuscitation. The serum osmolality declined gradually from 387 to 329 mOsm/kg over the first 48 hours (an average rate of 1.2 mOsm/kg/hour), with close monitoring and controlled correction of both sodium and osmolality. Despite metabolic improvement, coma persisted. Brain magnetic resonance imaging (MRI) on day three demonstrated high-signal intensities on diffusion-weighted imaging (DWI) in the right lateral amygdala and left thalamus, consistent with extrapontine myelinolysis (EPM) (Figure 1).

**Figure 2:**
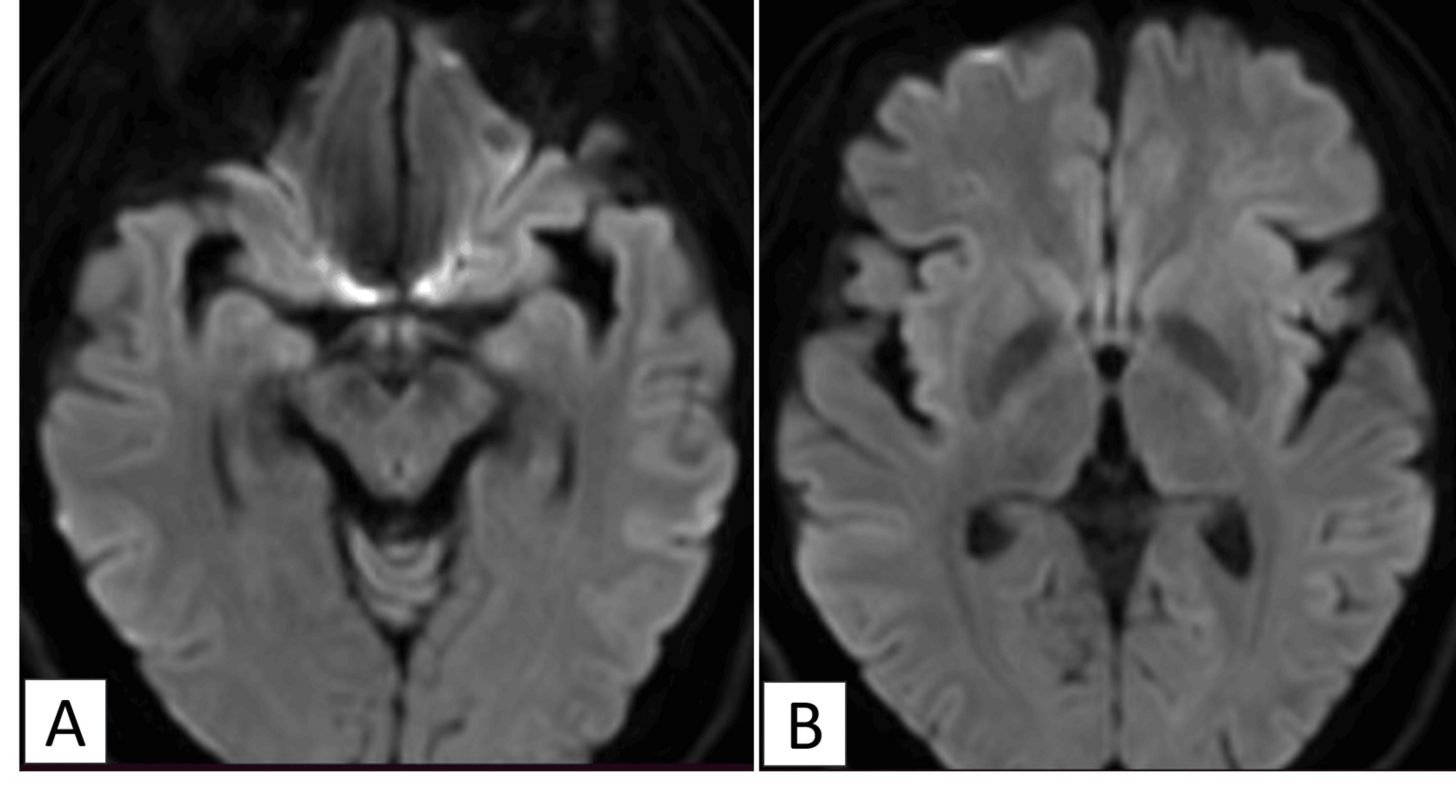
Case 1: Follow-up axial diffusion-weighted images (DWI) on day 214 The image demonstrates complete resolution of the previously noted hyperintense lesions in the (A) right lateral amygdala and the (B) left thalamus.

Electroencephalography (EEG) on day four revealed a burst suppression pattern. Levetiracetam was administered from days four to seven as a precaution, but no further seizures were observed. Due to an increase in creatine kinase (CK), possibly related to the medication, levetiracetam was discontinued. By day nine, a repeat EEG demonstrated an improvement in the burst suppression pattern. With supportive care and rehabilitation, the patient's modified Rankin Scale (mRS) improved from 5 at admission to 3 at transfer on day 59; extubation occurred on day 13, and oral intake resumed on day 45. A follow-up MRI on day 214 showed complete resolution of the previous lesions (Figure 2), and her final mRS was 2.

Case 2

A 62-year-old Japanese man with type 2 diabetes, hypertension, and dyslipidemia was transferred for persistent altered consciousness after four days of malaise and anorexia. He had been intubated at the referring hospital. On arrival, he was sedated with propofol (GCS E1VTM4), hypotensive (76/54 mmHg), and hypothermic (30.4 °C). Initial laboratory tests revealed severe hyperglycemia (1,277 mg/dL), hypernatremia (Na 149 mmol/L; corrected sodium 177 mmol/L), and marked hyperosmolality (421 mOsm/kg), along with metabolic acidosis (pH 7.03; bicarbonate 9.2 mmol/L). Serum β-hydroxybutyrate was 11.8 mmol/L (11,842 μmol/L), confirming significant ketoacidosis. Based on these findings, the patient was diagnosed with mixed HHS and DKA. Additionally, SARS-CoV-2 PCR was positive.

**Figure 3:**
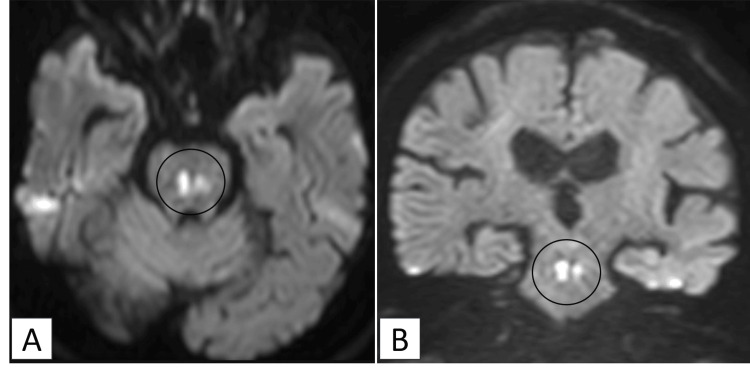
Case 2: Diffusion-weighted images (DWI) on day seven The axial view (A) and coronal view (B) both demonstrate a distinct, point-like hyperintense lesion in the central pons (circles).

In the ICU, he received fluids and intravenous insulin. Serum osmolality declined from 421 to 350 mOsm/kg over 72 hours (an average rate of 1.0 mOsm/kg/hour), with careful monitoring and controlled correction of both sodium and osmolality. After resolution of DKA and hypernatremia, he was extubated on day seven but remained nonverbal. A brain MRI performed the same day revealed a distinct pontine hyperintensity on DWI, consistent with central pontine myelinolysis (CPM) (Figure 3).

Neurological function improved steadily: he was alert and communicative by day 15, transferred to rehabilitation on day 22 with mRS 3, and discharged home on day 31 with mRS 1 (indicating minimal disability). Following his discharge, he was followed up by his primary care physician, and no new neurological symptoms were reported. Table 2 presents a detailed comparison of the clinical severity and outcomes for both cases.

**Table 2:** Clinical course and neurological outcomes for both cases The timeline of key clinical events (intubation, extubation, oral intake, and transfer/discharge), daily Glasgow Coma Scale (GCS), and modified Rankin Scale (mRS) scores from admission through follow-up are shown. Abbreviations: GCS, Glasgow Coma Scale; mRS, modified Rankin Scale; ICU, intensive care unit; DKA, diabetic ketoacidosis; ODS, osmotic demyelination syndrome; MRI, magnetic resonance imaging.

Parameter	Case 1	Case 2
Initial Severity Markers		
Initial Glucose (mg/dL)	2398	1277
Initial Serum Osmolality (mOsm/kg)	387	421
Initial GCS Score	E1V1M1 (3)	E1VTM4 (5T)
Outcome Measures		
mRS at Admission	5	5
mRS at Discharge (to rehabilitation hospital)	3 (Day 59)	3 (Day 22)
mRS at Final Follow-up	2 (Day 214)	1 (Day 31)
Days to Extubation	13	7
Days to Oral Intake	45	10
Days to Independent Ambulation	43 (with walker)	16 (unassisted)
Length of Hospital Stay (days)	59	22
Final Neurological Status	Near-complete recovery	Complete recovery

## Discussion

In this report, we describe two cases of ODS that developed in the context of HHS, a rare but serious complication. A key aspect of this report is the complexity of sodium abnormalities in HHS: in Case 1, the patient presented with profound hyponatremia on admission, but after correction for hyperglycemia, the "corrected sodium" was in the hypernatremic range. This highlights the importance of considering both measured and corrected sodium values when evaluating osmotic risk, as both can contribute to brain injury in HHS. The most distinctive aspect of this report is its head-to-head comparison of a hyponatremic and a hypernatremic ODS case occurring under comparable degrees of hyperosmolality, an approach that, to our knowledge, was not previously described in the literature. Despite these differences, both patients developed ODS, as diagnosed by MRI, with one case of EPM and the other of CPM. Notably, the rate of serum osmolality correction in both cases (1.2 and 1.0 mOsm/kg/hour, respectively) was highly cautious, remaining well below the conservative upper limit of 3 mOsm/kg/hour recommended by modern guidelines to prevent iatrogenic complications [16]. Both serum osmolality and sodium were carefully monitored and corrected at a controlled rate throughout treatment. This cautious rate of correction strongly suggests that the severe and sustained hyperosmolarity inherent to HHS itself, rather than the therapeutic intervention, was the principal trigger for the demyelinating process.

The timing of ODS development in our patients provides crucial insight into its underlying etiology. Classically, the neurological symptoms of ODS appear several days after the onset of corrective therapy, with characteristic MRI findings typically emerging one to two weeks following the initial metabolic insult [1,17]. While we cannot completely rule out the contribution of rapid or large fluctuations in sodium and osmolality, especially in the context of severe hyperglycemia, both patients were managed with correction rates that were well below the guideline-recommended thresholds. The clinical trajectories suggest a different primary mechanism. Neither patient demonstrated acute neurological deterioration following initiation of therapy; instead, both exhibited gradual improvement in consciousness. Furthermore, ODS was detected by MRI relatively early in the course of hospitalization, on day three in Case 1 and day seven in Case 2. This timeline supports the hypothesis that the demyelinating process was already underway prior to admission, likely initiated by the profound and sustained hyperosmolar state due to uncontrolled hyperglycemia and dehydration before medical intervention.

Several non-mutually exclusive mechanisms have been proposed to explain how hyperosmolar states induce ODS. A rapid increase in plasma osmolality can cause direct injury to astrocytes, which are crucial components of the blood-brain barrier. Disruption of this barrier permits the influx of inflammatory cytokines into the central nervous system, where they may damage oligodendrocytes, the cells responsible for producing and maintaining myelin sheaths, ultimately resulting in demyelination [18]. Another proposed mechanism is that hypertonic stress causes disruption of endothelial cells and the blood-brain barrier, resulting in plasma leakage and vasogenic edema. This process may contribute to the demyelinating cascade [19,20]. These changes preferentially affect brain regions rich in oligodendrocytes and myelinated fibers, including the pons, basal ganglia, and cerebellum, which is consistent with the lesion distribution observed in our cases.

Our case series adds to the growing body of literature on ODS occurring in the context of HHS. Although HHS-induced ODS is rare, the clinical presentations in our patients, with altered mental status being the primary presentation, are consistent with those described in previous reports [4,21]. It is noteworthy that, although the initial measured sodium levels differed (hyponatremia vs. hypernatremia), both patients experienced significant hyperosmolar stress and ultimately had corrected sodium levels in the hypernatremic range. While ODS has been documented in normonatremic or hypernatremic hyperglycemic states [5,13], presenting a hyponatremic case alongside a hypernatremic case underscores the principle that extreme hyperosmolality is the principal pathogenic factor, irrespective of the initial sodium concentration.

Furthermore, the early detection of ODS on MRI, on days three and seven in our patients, is notable, as some reports have described delays in the appearance of radiological abnormalities [17]. The divergent recovery trajectories observed in our patients, quantitatively detailed in Table 2, reflect the variable outcomes reported in the literature. The patient with CPM (Case 2) exhibited a more rapid and more complete recovery, evidenced by a shorter acute hospital stay (22 vs. 59 days) and a better final functional outcome (modified Rankin Scale score of 1 vs. 2). This supports the hypothesis that prognosis may depend more on the lesion location and extent than on the initial metabolic derangements [22].

We acknowledge several limitations in this report. The primary limitation is its nature as a retrospective case series, which precludes the establishment of causality and limits the generalizability of our conclusions. In addition, the complexity of sodium disturbances in HHS and the potential for both measured and corrected sodium to contribute to osmotic stress present challenges in categorizing cases solely by their initial sodium values. While the clinical evidence points toward pre-hospital hyperosmolality as the trigger, we cannot entirely dismiss the potential contribution of osmotic shifts during therapy. Another consideration is the confounding influence of SARS-CoV-2 infection in the second case, given the virus's association with neurological symptoms. Finally, the diagnosis was reliant on neuroimaging, as is typical in such cases, rather than the current gold standard of brain biopsy.

## Conclusions

The two cases presented here underscore that ODS is a critical, albeit rare, complication of HHS, likely triggered by the severe hyperosmolar state itself. Clinicians managing HHS must maintain a high index of suspicion for ODS when neurological deficits persist or remain unexplained, regardless of both measured and corrected sodium levels or adherence to recommended correction rates. Given the variable timing of ODS onset, clinicians should maintain a low threshold for obtaining timely MRI in such cases, as this may be instrumental in establishing the diagnosis. These cases highlight the clinical importance of evaluating both measured and corrected sodium levels, as well as the overall osmotic burden, when assessing ODS risk in patients with HHS.
